# Birthplace choices: what are the information needs of women when choosing where to give birth in England? A qualitative study using online and face to face focus groups

**DOI:** 10.1186/s12884-017-1601-4

**Published:** 2018-01-08

**Authors:** Lisa Hinton, Carol Dumelow, Rachel Rowe, Jennifer Hollowell

**Affiliations:** 1 Health Experiences Research Group, Department of Primary Health Care Sciences, University of Oxford, Oxford, OX2 6GG UK; 2 Policy Research Unit in Maternal Health and Care, National Perinatal Epidemiology Unit, Nuffield Department of Population Health, University of Oxford, Old Road Campus, Headington, Oxford, OX3 7LF UK

**Keywords:** Planned place of birth, Choice, Decision-making, Information, Internet, Midwives, Pregnancy, Qualitative data, Focus groups

## Abstract

**Background:**

Current clinical guidelines and national policy in England support offering ‘low risk’ women a choice of birth setting. Options include: home, free-standing midwifery unit (FMU), alongside midwifery unit (AMU) or obstetric unit (OU). This study, which is part of a broader project designed to inform policy on ‘choice’ in relation to childbirth, aimed to provide evidence on UK women’s experiences of choice and decision-making in the period since the publication of the Birthplace findings (2011) and new NICE guidelines (2014). This paper reports on findings relating to women’s information needs when making decisions about where to give birth.

**Methods:**

A qualitative focus group study including 69 women in the last trimester of pregnancy in England in 2015–16. Seven focus groups were conducted online via a bespoke web portal, and one was face-to-face. To explore different aspects of women’s experience, each group included women with specific characteristics or options; planning a home birth, living in areas with lots of choice, living in areas with limited choice, first time mothers, living close to a FMU, living in opt-out AMU areas, living in socioeconomically disadvantaged areas and planning to give birth in an OU. Focus group transcripts were analysed thematically.

**Results:**

Women drew on multiple sources when making choices about where to give birth. Sources included; the Internet, friends’ recommendations and experiences, antenatal classes and their own personal experiences. Their midwife was not the main source of information. Women wanted the option to discuss and consider their birth preferences throughout their pregnancy, not at a fixed point.

**Conclusions:**

Birthplace choice is informed by many factors. Women may encounter fewer overt obstacles to exercising choice than in the past, but women do not consistently receive information about birthplace options from their midwife at a time and in a manner that they find helpful. Introducing options early in pregnancy, but deferring decision-making about birthplace until a woman has had time to consider and explore options and discuss these with her midwife, might facilitate choice.

**Electronic supplementary material:**

The online version of this article (10.1186/s12884-017-1601-4) contains supplementary material, which is available to authorized users.

## Background

It is current national policy in England that every woman should be able to choose the most appropriate place and health professional for her care “based on her wishes and cultural preferences and any medical and obstetric needs she and her baby may have” [1]. The 2014 NICE guideline on intrapartum care for healthy women and babies [2] recommends that women be advised that they may choose any birth setting including at home, in a freestanding midwifery unit (FMU), alongside midwifery unit (AMU) or an obstetric unit (OU).

This recommendation was based in large part on the findings of the Birthplace in England cohort study which was published in 2011 [3]. This study demonstrated that, for healthy women with straightforward pregnancies, planned birth in a midwifery unit was safe for the baby, and that planned home births were safe for women having a second or subsequent baby, but with some increased risk to the baby for women having a first baby. Planning birth at home or in a midwifery unit was also associated with a reduced chance of the woman having an intervention during labour or birth, including augmentation, epidural/spinal analgesia and an instrumental or caesarean birth.

The 2014 NICE guideline states that *“It is important that the woman is given information and advice about all available settings when she is deciding where to have her baby, so that she is able to make a fully informed decision”* and gives recommendations about the information which should be provided to women. The guideline includes a table of intervention and transfer rates and perinatal outcomes for each birth setting, which it is suggested should be discussed with women planning place of birth. However, the guideline acknowledges that there is little evidence on how this information should be presented to women and how the provision of information affects women’s decision-making about place of birth.

In England, the largest of the four countries of the UK, the provision of midwifery units, particularly AMUs within hospitals, has increased substantially [4] since these two publications. In 2013, 79% of women in England lived within 30 min’ drive of both an OU and a midwifery unit (AMU or FMU) [5]. More recently a national survey of women’s experiences of maternity care found that 41% of women were offered a choice of giving birth in a midwifery unit and 39% were offered the option of a home birth [6]. However, despite this apparent range of options, recent data show that the vast majority of women (87% in 2013) still give birth in an OU; the home birth rate is static at around 2.3% of births; [7] and although the number of FMUs has increased slightly in recent years, the proportion of women giving birth in FMUs in England remains static at around 2% [8].

While some women who plan birth in consultant-led OUs (see Table 1) may do so because of lack of choice in their area, [9] there is also evidence to suggest that most women currently consider that a ‘hospital’ with onsite medical facilities is the ‘normal’ and ‘safest’ place to give birth [10]. However, research has also shown that such views are not fixed and that the provision of information about alternative options may change women’s views and preferences [11]. The extent to which most women are aware of differences between types of setting for birth, or are given information about their possible options, is unclear [5].

**Table 1:** Terms used to describe birth settings (adapted from Rowe et al., [51])

Obstetric unit (OU) (also called a ‘labour ward’ or ‘delivery suite’)	NHS maternity unit in which care is provided by a team, with obstetricians taking primary professional responsibility for women at higher risk of complications during labour and birth. Midwives care for all women in an OU and take primary responsibility for ‘low risk’ women. Diagnostic and treatment medical services including obstetric, neonatal and anaesthetic care are available on site, 24 h a day.
Alongside midwifery unit (AMU)	NHS maternity unit for ‘low risk’ women in which midwives take primary professional responsibility for care. Diagnostic and treatment medical services, including obstetric, neonatal and anaesthetic care are available, should they be needed, in the same building, or in a separate building on the same site. Transfer will normally be by trolley, bed or wheelchair.
	Admission to an AMU may be based on an ‘opt-in’ policy whereby ‘low risk’ women choose to plan birth in the AMU, or ‘opt-out’ whereby the AMU is the default option for ‘low risk’ women.
Freestanding midwifery unit (FMU)	NHS maternity unit for ‘low risk’ women in which midwives take primary professional responsibility for care. General Practitioners may also be involved in care. Diagnostic and treatment medical services, including obstetric, neonatal and anaesthetic care, are not immediately available but are located on a separate site should they be needed. Transfer will normally be by car or ambulance.
Home birth	Planned birth in the woman’s own home, where care is provided by an NHS or independent midwife. If diagnostic and treatment medical services are required, transfer will normally be by car or ambulance.

Against this backdrop, the *Birthplace Choices* project, [12] was designed to inform policy on ‘choice’ in relation to childbirth. A systematic literature review conducted in early 2015 as part of the project [13] did not identify any evidence relating to UK women’s experiences of choice and decision-making in the period after publication of the Birthplace findings in 2011. The Birthplace study provided robust evidence about the safety of different birth settings in England [14]. The study prompted substantial changes to online NHS patient information for pregnant women [15] and also changes to the recommended advice to be given to women about choosing where to give birth, with midwifery units and home births (for multiparous women) becoming the recommended setting for ‘low risk’ women [2]. To provide evidence about women’s experiences in the wake of these changes, and also to inform the design of a discrete choice experiment (DCE) which forms part of the Birthplace Choices project, we undertook qualitative research, using online focus groups (which included both online ‘chat’ and message boards) and one face-to-face focus group with women across England. This component of the *Birthplace Choices* project aimed to explore: (a) the factors that are important to women when making a choice between different settings for birth; (b) the attributes of maternity services which women particularly value; (c) the services that an NHS trust needs to provide in order to offer women a realistic choice of home birth; (d) the effect of travel time and distance on women’s choices; and (e) how women access and evaluate information about birth place options. In this paper we report on findings relating to women’s information needs and preferences, including their knowledge of the birth place options available to them, how they gathered information about their options from different sources and their views on the information they would like to receive from midwives. Findings relating to other objectives will be reported in a separate paper relating to the development and piloting of the Birthplace Choices DCE.

## Methods

This study was a qualitative focus group study, with eight groups conducted either online or face to face, with women in the last trimester of pregnancy in England.

### Ethics approval

The study methods and documentation were reviewed and approved by University of Oxford Central University Research Ethics Committee (CUREC). The study received CUREC approval to offer a shopping voucher (for £25) to participants to thank them for their time taking part in the study.

### Stakeholder consultation

During the design phase of the project two consultation meetings were held with User Group representatives and other stakeholders (see acknowledgements) to ‘brainstorm’ issues that the project might address and to develop and test a conceptual model of access to choice (which has been used and reported elsewhere [4, 13]) This input informed decisions about the composition of the focus groups and the topics included in the discussion guide (Additional file 1).

### Recruitment and sampling

To explore different aspects of women’s experience, each group included women with specific characteristics or options as identified by our knowledge of the literature and feedback during our stakeholder consultation. The groups were women:

- planning a homebirth (group 1),
- living in areas with lots of choice (group 2),
- living in areas with limited choice (group 3),
- who were first time mothers (group 4),
- living close to an FMU (group 5),
- living in opt-out AMU areas (group 6),
- living in socioeconomically disadvantaged areas (face to face) (group 7),
- planning to give birth in an OU (group 8).

Each focus group included up to 12 women who were in their third trimester of pregnancy. Women were included if they were expecting to have a spontaneous vaginal birth, had a low risk pregnancy with no complications, were 18 years and over and lived in England. Our criteria therefore excluded women who were expecting to have a Caesarean birth and other groups of women who would normally be advised to give birth in an OU, such as those with medical risk factors or current pregnancy complications. Details of participants are shown in Table 2 (with additional details in Additional file 2).

**Table 2:** Characteristics of focus group participants

Group/ name	Age	IMD quintile ^a^	Parity	Type of area/ region	Option interested in/ chose
Group 1 – Planning a Homebirth					
Ann	32	3rd	Primip	City, North East	Homebirth
Anita	32	1st	Multip	Town, South West	Homebirth
Angela	34	1st	Multip	City, South West	Homebirth
Amy	28	5th	Primip	City, East Midlands	Homebirth
Amelia	33	5th	Primip	London	Homebirth
Alison	30	1st	Multip	Town, South East	Homebirth
Amanda	38	2nd	Multip	Town, South West	Homebirth
Alexa	28	4th	Primip	London	Homebirth
Abigail	31	1st	Multip	Town, South East	Homebirth
Agnes	30	2nd	Primip	Town, North West	Homebirth
GROUP 2 – Living in areas with lots of choice					
Jenny	30	2nd	Primip	City, North West	AMU
Julie	NK	3rd	Primip	London	OU
Jude	28	3rd	Multip	Town, East of England	AMU
Janice	34	4th	Primip	London	AMU or FMU, possibly home
Joyce	26	2nd	Multip	Town, South West	Homebirth
Jackie	28	3rd	Primip	Town, South West	Homebirth
Joanna	42	3rd	Primip	City, West Midlands	OU
Jean	24	3rd	Primip	Town, East of England	OU
GROUP 3 – Living in areas with limited choice					
Rachel	34	2nd	Primip	Town, South West	Homebirth, possibly FMU
Rebecca	30	2nd	Primip	Town, West Midlands	Homebirth, possibly AMU
Ruby	32	2nd	Primip	Village, North West	OU
Rosie	34	2nd	Multip	Town, South East	Homebirth
Rita	35	2nd	Primip	Village, South East	Homebirth
Roberta	32	1st	Primip	Village, South East	FMU
Rona	38	3rd	Multip	Town, South West	Homebirth
GROUP 4 - First time mothers					
Mandy	31	1st	Primip	Town, South East	AMU
Maria	33	5th	Primip	London	FMU
Mabel	36	2nd	Primip	Town, East of England	AMU (27 miles, not nearest)
Mina	33	4th	Primip	City, Yorkshire & Humber	OU/AMU
Mae	27	1st	Primip	Village, South East	AMU or FMU
Maggie	32	5th	Primip	Town, North East	OU
GROUP 5 – Living close to an FMU					
Kirsten	26	4th	Multip	Town, South West	FMU
Karolina	35	5th	Multip	City, South West	FMU
Keira	35	5th	Multip	City, South West	AMU or FMU
Katrina	NK	5th	Multip	Town, South East	AMU or FMU
Kylie	27	1st	Multip	Town, South East	AMU
Kathryn	34	2nd	Primip	City, South West	AMU
Kim	36	1st	Primip	Village, South East	FMU
Kerry	30	2nd	Primip	Village, South East	AMU
Kate	35	1st	Primip	Village, North West	FMU
GROUP 6 – Opt-out AMU areas					
Clare	32	1st	Primip	City, Yorkshire & Humber	OU
Charlotte	34	4th	Primip	City, West Midlands	AMU
Chloe	39	3rd	Multip	City, North West	AMU
Courtney	32	4th	Primip	City, North West	AMU
Clara	35	5th	Primip	City, North West	AMU
Chantel	38	4th	Multip	Town, East Midlands	OU
Carmen	29	2nd	Primip	City, South West	AMU
Carrie	31	4th	Primip	City, South West	AMU
Cath	32	NK	Primip	Village, East Midlands	AMU
Caroline	32	4th	Primip	City, North West	AMU
Carla	NK	1st	Multip	City, North West	OU
GROUP 7 (face to face) – Living in disadvantaged area^b^					
		Ethnicity			Booked for birth in:
Yasmin	NK	Black African	Primip	East London	OU (not low risk)
Yana	NK	Bengali-speaking	Primip	East London	OU (not low risk)
Yadavi	NK	Bengali-speaking	Multip	East London	OU
Yavi	NK	Indian	Multip	East London	OU (not low risk)
Yelena	NK	Eastern European	Multip	East London	FMU
Yusra	NK	NK	Multip	East London	AMU
Yihana	NK	Black African	Multip	East London	AMU
GROUP 8 - Planning to give birth in an OU					
Brenda	23	5th	Primip	City, North West	OU
Barbara	29	4th	Primip	City, North West	OU
Beatrice	35	1st	Multip	Town, South East	OU or Homebirth
Bev	28	2nd	Multip	Town, South East	OU or Homebirth
Beverley	35	4th	Primip	City, South West	OU
Bobbi	29	2nd	Multip	City, South West	OU
Bree	33	1st	Multip	Town, South East	OU
Belinda	32	4th	Multip	Town, South East	OU
Bridget	35	3rd	Multip	City, South West	OU
Bonnie	39	4th	Multip	London	OU
Betty	25	2nd	Primip	Town, South West	OU

^a^ Index of Multiple Deprivation (IMD) quintile: 1st = most disadvantaged, 5th = most advantaged

^b^ Participants recruited through local women’s support group and not pre-screened by the researchers

The web portal online chat was accessible to women who had a laptop or desktop computer. We also gave women the option of using an iPad and taking part in the discussion boards only. To be able to include as socio-economically diverse a sample as possible, we offered to reimburse women if they needed to use an internet café or library so that they could still take part if they did not have the technology at home. No one took up this offer.

Pregnant women were recruited from non-NHS settings such as antenatal classes, pregnancy yoga groups, libraries, children’s centres, national childbirth organisations, online pregnancy and parenting forums, personal contacts, and word of mouth. “Gatekeepers” (for example the organisers of a yoga group) were identified through online resources and were contacted by the researcher asking for help in publicising the study to recruit pregnant women for the focus groups, using word of mouth, handing out flyers and displaying posters. Women interested in finding out more were asked to contact the researcher (CD) directly. We also had a Birthplace Choices webpage providing details of the project for potential participants. If women were interested in taking part they were sent the participant information sheet and a consent form via the post or email and asked to complete the consent form before the focus group started. Women were given a shopping voucher after the focus groups to thank them for taking part in the study.

To ensure that we did not exclude women who did not have access to online technology, we held a face to face group with 10 women in Tower Hamlets, London, recruited through a local women’s group who support disadvantaged and vulnerable pregnant women.

### Focus groups

The eight focus groups were held across England between July 2015 and March 2016 with a total of 69 pregnant women in their third trimester. To maximise the geographical spread of women we could recruit, we used an online approach for seven of the eight groups. Focus groups were conducted online, using a specially designed (closed) web portal called “Birthplace Choices”. The web portal was designed and supported by the DIPEx Charity, which is closely linked with the Health Experiences Research Group as it publishes the Healthtalk website [16]. The staff has the expertise to provide the online support that we needed to create and host the web portal. Each focus group participant had access to the web portal through an individually assigned password. Only members of the research team and women participating in the specific focus group had access to the focus group discussions and the women were given anonymous identities (see Table 2). The online focus groups each lasted a week to give women the opportunity to participate flexibly. Each online group started with a live text-based web chat lasting 60 min where women were able to introduce themselves, get to know each other and initial questions were posed by the moderator. The online focus group finished at the end of the week with another live web chat, this time only 30 min. In between these two live chats, the women were invited to participate in discussion boards on which one of the researchers posted further questions. The online groups were moderated and facilitated by LH. The discussion boards were facilitated by CD. The extended time frame was intended to give the opportunity for participants to overcome the inevitable anonymity of an online platform. Having an initial live chat followed by several days to reflect on the questions posed meant that the women were able to get to know each other, and the researchers, build rapport and consider and reflect on the questions posed. The live chats and message boards elicited different responses; while the live chats were both quite busy, fast paced conversations, the message boards were an opportunity for women to reflect on their answers more slowly. The final live chat was a way for all participants to pick up on threads of conversation and for the researchers to pose final follow up questions that had emerged.

The face to face group was moderated by LH and was audio recorded. This group included women who did not speak English as their first language. An interpreter was present to assist when required.

Interview guides were designed for the first online web chat and initial discussion board questions, and for the face to face group, based on our knowledge of the literature and our discussions with stakeholders. The interview guides were amended slightly according to the focus of each group and developed iteratively as the study progressed. Questions for the second online chat for each group were informed by the issues raised by the women on the discussion board during the week.

### Analysis

Transcripts were created automatically from the data generated by the online groups. Transcripts from the face to face group were transcribed and translated (where required, most of the discussion was in English). All the data were entered into NVivo qualitative data analysis package [17]. A coding frame was developed by CD and LH (Additional file 3). They read a sub-sample of transcripts and conferred about the framework before each transcript was coded. A thematic analysis was conducted with key categories and themes identified using the ‘One Sheet of Paper’ (OSOP) method [18]. Pseudonyms were used during the online focus groups. Participants in the face to face group have been assigned pseudonyms (see Table 2).

## Results

The results presented here report on women’s knowledge of the birth place options available to them, how they gather information about their options and which sources they draw on. We also report on the information that women would like from their midwives.

### Knowledge of options available

Few women we spoke to had been aware early on in their pregnancy of the options they could choose from in their local area. Some knew about the options from previous pregnancies, or they had researched it themselves, or knew they wanted a home birth so had not researched the other options. For others, knowledge of their options increased as their pregnancy progressed. This knowledge was gained from a wide range of sources. Midwives were not the main source of information for women (see below).

Charlotte didn’t know she had more than one choice until she attended her local NCT classes. Belinda found out from another mum that she could choose a different hospital than the one closest to her. Bobbi learned through Facebook that she could choose another hospital.

“I literally found out today from some random Facebook comments that I *didn't* have to choose my local hospital. I still would, because of distance, but it might have been useful to know.” (Bobbi, Focus group 8, planning birth in an OU).

Reflecting on her knowledge of what choice she had in her first and second pregnancy, Katrina said, *“I wasn’t aware there was a choice with the first pregnancy. Second pregnancy feel more informed but perhaps that is because I know what to ask.”* (Katrina, Focus group 5, living close to an FMU).

Brenda, a first-time mum, talked about finding out about her birth options, “*I'm not sure if I'm supposed to be looking or they're supposed to tell me about my options but I normally research everything myself. I haven't had this conversation with the midwife as well.”* (Brenda, Focus group 8, planning birth in an OU).

Other women were not aware there was a choice or aware of all the options available to them. Some women did not know there was a choice at all. Some were not aware that they could choose other hospitals than the ones nearby them.

*“I wish I’d realised I had a choice to be able to research a bit, to feel more relaxed about our decision.”* (Katrina, Focus group 5, living close to an FMU).

Some were not aware that home birth was an option. A few did not know there was a difference between the AMU and an OU.

Finding out later in pregnancy about available options did lead to some women changing their decision to another option; often a less medical setting than the one they had originally chosen. But more information could also be used to reassure women that the choice they had made was the right one for them. When asked if she felt supported in her choice, Clara, (Focus group 6, opt-out AMU areas) said *“not really, it was much my own decision and the research I’ve made myself”.* By the end of their pregnancy many knew of all the options available to them and several said the area they lived in offered all the options they would like (close to FMU, planning homebirth, lots of choice, limited choice groups).

### How do women gather information about their options of where to give birth?

When deciding where to give birth, many women we spoke to had researched their birth options. Even if they knew what option they wanted, several still looked for ‘confirming’ information that helped them to feel more confident about their choice and sometimes to justify their decision to others.

Some women were offered tours of units in hospitals, and found these visits helpful and reassuring.

*“I've been on a hospital visit which made me feel a lot better about my choice.” (Caroline, Focus Group 6, optout AMU areas)*.

However, there were several women who said that their hospital either did not offer tours, or were too busy to allow women to look around.

“*We were told that unless you felt particularly anxious about the birth most hospitals (one exception) did not offer the opportunity to visit. I appreciate the challenge for the hospital in doing this but would have liked to have the opportunity to visit to help make an informed decision*.” (Cath, Focus Group 6, opt-out AMU areas).

Two different groups of women, those living in areas where there was less choice (e.g. near opt-out AMUs or where FMUs were not available) and those who were second or third-time mothers, did less research when making their birth place decisions. Some of these women said they did not do any research online about their birth options but they talked to family and friends.

“*I don’t think I did much research first time nor second nor this time lol! I just kinda feel like this is my third I'll just go with the flow! I know that the only options around here are hospital or home!”* (Bev, Focus group 8, planning birth in an OU)

### Information sources

Women drew on multiple information sources when making their decision about where to give birth. The Internet, friends’ recommendations and experiences, antenatal and birth preparation classes and their personal experiences of birth were the main sources used. Some women we spoke to had also gained information from their midwife. But this was mostly not the main source of their information.

*“My midwife at [OU] gave me leaflets and information about the options and their website is good. I also researched online (there's a good tool that ‘Which?’ have for making your decision) and talked to friends. All of my friends have given birth either in labour wards or in midwife-led centres and that feels right for me.”* (Julie, first time mother, Focus group 2, lots of choice).

*i) The Internet* was used regularly by women to find more information about a range of factors when making their decision about where to give birth. Some used the Internet to look for the facilities available at their local maternity units. Some researched statistics on assisted births, caesarean rates, transfer rates and for evidence on the safety of different options by using the Internet. Websites such as NHS Choices, Which? Birth Choice, NICE guidelines, Association for Improvements in the Maternity Services (AIMS), Positive Birth Movement, the National Childbirth Trust (NCT) website were popular.

*“I must say that my husband and I found the matter of choosing where to give birth at the very beginning very daunting (this is our first child) and it was not clear that it was a decision that could change later in the pregnancy till about a month ago. For that we talked briefly to friends but mostly based our decision to go to the [hospital A] from various online sites about differences of nearby options and varying stats about the birthing units like interventions. About a month ago when it became clear we could change location if we liked we spoke to family and friends and also used the NHS reviews page to gauge opinion between the [hospital B] and the [hospital A] hospitals. We have decided to go with the [hospital A]. I know people who birthed at the [hospital A] and they had mixed reviews. I know no one who has birthed at the [hospital B].”* (Maggie, Focus group 4, first time mothers).

Some of the women in the opt-out AMU group and the OU group said they had not looked for any further statistics and when prompted some were not aware that statistics on interventions existed.

*“No, didn't know that information existed.”* (Cath, Focus group 6, Opt-out AMU).

*“no i havent looked at the intervention rates, is it available for us to view?”* (Carrie, Focus group 6, Opt-out AMU).

The Internet was also used to find out about other women’s experiences of birth. Netmums and Babycentre.co.uk were a popular source for this type of information. Rachel had read about birth stories online or watched YouTube videos about birth. Some women choosing a home birth had joined social media groups such as the Positive Birth Movement.

*ii) Friends* were a very important source of information and their experiences of birth were very influential on the decisions that women made. They were considered to be a more reliable source of information by some women who said they would be wary of looking at Internet forums for people’s experiences because they may have an agenda. Alexa was planning a home birth. She was a first time mother, herself a midwife and had been exposed to lots of women’s birth experiences.

*“Because birth is such an emotive experience, hearing people's experiences directly has had more of a meaningful impact than what I have read and the way they tell the story, as well as what they say, has been influential. When women I've looked after or friends speak passionately and positively about their birth experiences, wherever they had their baby, I always feel inspired!”* (Alexa, Focus group 1, Home birth).

Conversely Janice said she had avoided talking to friends in too much detail about their birth experiences because many had had ‘tricky births’. Friends provided information on their experiences of different types of birth and their experiences at local maternity units. Several women we spoke to said that their friends’ experiences (either positive or negative) at a local maternity unit had influenced their decision about which maternity unit to choose.

“*Good reputation and feedback from friends……People we know and their experience*”. (Clara, Focus group 6, opt-out AMU areas).

*“Real life experience, and reliable information”* (Mina, Focus group 4, first time mothers).

*“More discussion with my midwife halfway through the pregnancy would have been helpful, as I mentioned my midwife is very much about the box ticking and rushing through the appointments without much time for my inconvenient questions! What I have learnt has been through friends or NCT.” (*Mandy, Focus group 4, first time mothers).

But Cath explained that her friends had positive experiences at all the local maternity units so friends’ experiences weren’t all that helpful in her decision-making.

Sometimes friends’ stories of birth in hospital had influenced women to consider other options and had put them off having a hospital birth. Julie had friends who had recently had midwife-led or OU births and she said this seemed the right thing for her too. Jackie, Julie and Kerry had doctor friends who were very discouraging of anything other than a hospital birth and for Kerry and Julie, these views had been an important factor in their decision-making.

*“I haven't felt discouraged by anyone. Family and health professionals have nudged me towards a hospital birth, but I'm with them.”* (Bree, Focus group 8, planning birth in an OU).

*iii) Classes*. Antenatal/birth preparation classes and pregnancy exercise classes were also an important source of information for some women about the range of birth place options available to them, although they are generally attended later in pregnancy. Mina and Rosie found out that home birth was an option in their areas and Joanna and Badger learnt about the differences between the AMU and the OU. Roberta learnt through her NCT classes that she could change her mind about her choice of birth place right up until she went into labour.

*“We have been going to NCT classes and that is where I found out that there's a difference between the birth unit and labour ward. I also found out there that it makes a difference in pain relief, for instance you can't have an epidural in the unit but would be transferred to the ward.”* (Mabel, Focus Group 4, first time mothers)

*“i learnt more about that at nct - my midwife jus handed me a bunch of leaflets and sent me on my way to read up.”* (Mandy, Focus group 4, first time mothers).

*iv)* Another important source of information for women we spoke to was their *personal experience* of birth, whether this was their own experience of giving birth or their mother’s or family members’ experiences. Women’s own previous birth experiences (whether negative or positive) shaped their decisions for their choice of birth place this time. Some of the women we spoke to had been brought up in a family environment where home birth was the norm and so they had not considered any other option when deciding where to give birth themselves. These women used local home birth support group or positive birth movement groups to learn more about having a home birth in their local area.

*v) Midwives* Women were asked about the information provided by their midwives. Surprisingly, given national guidance, their midwife was not a main source of information for women about birth choice options. Some women said that their midwife appointments felt rushed and there was not time to ask questions, with the focus on assessing physical wellbeing and little chance to talk about how they were feeling or their choices around birth. Some said that their midwives had asked them at the booking appointment where they wanted to give birth without giving them enough explanation of the options available to them. Mabel (Focus Group 4, first time mothers) explained, *“The staff are very nice, but they tick boxes and you’re out the door.”*

*“I don't think midwives are aware that the whole birthing process is a mystery to new mums, since they do it so often and probably can't imagine what it is like when you've never “been there”.”* (Beatrice, Focus group 8, planning birth in an OU).

*“I don’t feel like I was given much information about all my options – just rolled out the options like a list.”* (Jude, Focus group 2, lots of choice).

A few women felt they had made uninformed decisions as a result of inadequate information from their midwife. Mina (Focus group 4, first time mothers) said she had realised that she would prefer somewhere more relaxed and non-medical, but did not feel there was anywhere close, “*to be honest if I was starting this again with what I know now, I would probably explore the midwife led unit more”.*

*“I was asked at my very first midwife appointment at 8 weeks. I had the choice of 4 hospitals […] and was asked to pick one. Having no real knowledge of the others I picked the one I knew. I'm sure I could switch now but I've got my head round where I'll be going, had a look round, explored parking options etc! I feel I know where I am with the [hospital] and that it’s a bit late to be changing now.”* (Mina, Focus group 4, first time mothers).

*“I don’t feel there is enough information either and I didn’t realise until later on in my first pregnancy I could choose other hospitals as my closest one does not have a midwife led unit. Information about other hospitals seems hard to find out. It would be great to be given more information earlier on and told when tours are on too in hospitals.”* (Abigail, Focus group 1, planning a home birth).

Other women had felt that they did have time to discuss their options throughout their pregnancy. But, in some cases, women said they only found out information because they asked questions. A second-time mother (Joyce) said she had to ask about a home birth, it had not been presented as an option.

*“If I hadn't mentioned home birth I don’t think anyone would have presented it to me as an option, we always get asked which hospital we want to go to”* (Joyce, Focus group 2, lots of choice).

Unlike some of the other women we spoke to, Bree had been told by her midwife she could choose another local hospital to give birth if she wanted to.

“*I'm planning [Hospital A] but told by my mw that we could go to [Hospital B] if we want - apparently its lovely*” (Bree, Focus Group 8, planning birth in an OU).

A few women knew which birth option they wanted and so they did not feel the need to have any further information from their midwife. Some said they thought this was why their midwife did not talk about any other options with them. Often this was the case for women who had given birth before.

### Information women would like from midwives

Many women said they would like their midwives to provide information about all the options available to them including further details about each option. Importantly, women wanted something they could take away with them, mull over and talk to others about and then have a discussion with their midwife at a later time in their pregnancy about their decision.

Information was needed early on in pregnancy about all the available options, with more detail about the individual options. Many wanted time to learn about all their options before making a decision. Some suggested a leaflet or booklet to be given at their first meeting so that they could think about their options, possibly discuss with partner, and then discuss at a later appointment with the midwife. Rona suggested the leaflet could have links to where women could do their own research.

*“I agree, a leaflet was a good source of information to me first time round. However, I found out through my hypnobirthing classes about being able to research statistics etc on transfer rates, assisted births and caesareans etc. So perhaps with the leaflet a couple of websites or pointers as to where people can go to do some research about their options would also be useful. I think right at the start, at the booking appointment or even in the Drs Surgery, would be the best time. You can always keep it in your notes for when you are ready to think about where you want to give birth.”* (Rona, Focus group 3, limited choice).

*“I was asked at my booking appt - when things were still so new and unknown - where I wanted to give birth before I had even thought that I had a choice so I went away after that and researched things on my own and then confirmed that I wanted to be in the midwife-led centre, ideally. So the midwife didn't really have an influence. I think it would have been better if she had said - “there are a number of options at [hospital], here’s a leaflet, you don’t have to say what you want now - let’s talk about it next time””* (Julie, first time mother, Focus group 2, lots of choice).

*“It would be nice to have a brief group session led by the community midwives that gives guidance on what is available at each unit in the area. I picked a hospital that was a specialist maternity hospital thinking that would probably be the best. What I really want is a water birth and it wasn't until I was 35 weeks I discovered that there are only two pools at this hospital, while there are ten pools available at the hospital which would have been my second choice*.” (Barbara, Focus group 8, planning birth in an OU).

Some would like detailed information to be provided including statistics for each option on facilities and medical expertise available, rates of intervention, unplanned caesareans and the pros and cons of each option. Others would be happy with a written outline of the options available and a list of websites where they could find out further information for themselves. Forums and group meetings were suggested as another way of disseminating this information, as women we spoke to recognised the limited time that midwives had to spend with them.

*“I think we have to take responsibility for our own education sometimes. I did NCT and it was great but there are ante-natal classes out there for free. I don't necessarily think midwives should have to allocate their limited time to giving 1-2-1 sessions on options and possible eventualities when it can be delivered to a larger group of those who take the initiative to find out.”* (Bridget, Focus group 8, planning birth in an OU).

Views on the best time for midwives to provide this information varied.

*“It's a difficult one as too much information can be an overload and not enough can make people feel uncertain. Parents should maybe be asked at what stage they want the information and how much information they want.”* (Bev, Focus group 8, planning birth in an OU).

Some women suggested a detailed discussion would be best at about 20 weeks. Amelia (Focus group 1, planning a home birth) said this *“would be early enough to do proper research and consideration but far enough in that you are starting to think about birth.”* A few women considered 36 weeks to be a better time for them to have this discussion.

Despite variation on when is the best time to give information, one point was clearly made, that women should have the option to discuss preferences throughout their pregnancy. Some felt it was important for the midwife to give women the opportunity to ask questions and discuss options at subsequent appointments – not just handed a leaflet, because getting a lot of information can be overwhelming when first pregnant.

“*I think setting out the choices at the beginning and then an ongoing discussing throughout the pregnancy to talk about any questions or changes of mind would be best. I don't think any choices around birth should be set in stone at any point really. The whole process is fluid and evolves so really everyone being open to discussing different options throughout would be ideal.”* (Bobbi, first time mother, Focus group 8, planning birth in an OU).

### Evaluating information

Women were forthcoming about the wide range of information sources they drew on, but they did not expand in very great detail about how they evaluated those different sources. Some women said they were wary of relying on online chat or internet sites like Mumsnet, “*as people often go online with an agenda.”* (Maria, Focus group 4, first time mothers), others sought out the advice of friends and “*reliable information*” (Mina, Focus group 4, first time mothers). But how they decided what information was or was not reliable did not emerge from these data.

*“Informations are very good and easy to evaluate however I feel much better to speak with midwife who in my opinion should be more competent. I haven't looked at any statistics[……] For me talking to my friends was more important than statistics.”* (Clara, Focus Group 6, Opt-out AMU).

Some women explained that they trusted their friends as a source of information because their opinions were “*Non biased*” (Mandy, Focus group 4, first time mothers) and based on their personal experiences: *“Because they’ve actually experienced it.”* (Mae, Focus group 4, first time mothers).

Some women mentioned seeking out statistics “*I did a lot of research on the internet for statistics.”* (Rona, Focus group 3, limited choice) and some explained how they used “statistics” and “evidence” to support their choices but did not talk about how they evaluated the information.

## Discussion

This is the first large-scale study to our knowledge to seek qualitative insights into how women make decisions about where to give birth since the NICE intrapartum care guidelines were updated in 2014. In the findings reported here we focus on women’s information needs and information sources which is something given considerable emphasis in the updated NICE guideline. In particular, those guidelines recommend that women should be told that they may choose any birth setting and should be given detailed information about the different birth settings available. This includes information about rates of intervention and serious adverse outcomes, and local statistics on access to a familiar midwife and one-to-one care, access to medical staff, access to pain relief and, for midwifery-led settings, the likelihood of transfer, the reasons why transfer might happen and the time it may take. Many of the women in our study were not told this information by their midwives and some were wholly unaware that statistics on intervention rates were available.

Our findings suggest that women draw on a *wide pool of information* sources to form their views. Hospital reputation, word of mouth and friends’ experiences at the maternity units were important factors in their own decision-making. The sources of information for making a choice about where to give birth that the women described using included: the Internet, friends’ recommendations and experiences, antenatal and birth preparation classes and their own personal experiences of birth. Their midwife was not the main source of information, with some saying their midwife appointments felt rushed and there was not time to ask questions. Women wanted to have the option to discuss birth preferences with their midwife throughout their pregnancy. Many women would like their midwives to provide information about all the options available to them including further details about each option. Importantly, women wanted something they could take away with them, mull over and talk to others about and then have a discussion with their midwife at a later time in their pregnancy about their decision. Nevertheless, although not reported here, some women commented that they felt supported in their choices by their midwives.

The results of our study should be considered in the light of a changing provision and information landscape. From the provision side, the availability of midwifery-led options, particularly AMUs, has expanded considerably in the past 5–10 years [4, 5]. This means that a greater proportion of women now potentially have access to both an OU and at least one midwifery unit (AMU or FMU) as well as the option of a home birth, although some, including some of our study participants, still may have only a limited choice available, for example between an OU and home birth. National survey data indicate that the proportion of women being offered birth choices by their midwife is increasing. The most recent nationally representative survey in 2015, in which over 20,000 women were surveyed after their birth, showed 41% reported that they had been offered a midwifery unit and 39% a home birth and 58% reported that they had received enough information from their doctor or midwife to help them decide where to give birth [6]. However, this leaves around 4 women in 10 not receiving sufficient information from their midwives or doctors.

### Discussion in light of other literature

Previous qualitative [19, 20] and quantitative studies [11, 21–24] from the early 1990s through to 2011 (reviewed more fully elsewhere [4]) found that women were not necessarily aware that choice existed or believed that the only available choices were between OUs. However, in some studies there were examples illustrating that some women were adequately informed and supported in their decisions by their health care professionals [11, 25, 26]. Previous studies also found that even where women were aware that alternative options were available their choices were sometimes blocked or discouraged by healthcare professionals [27–31]. The most recent study of women’s experience of birth place decision-making found that midwives were influential, but did not always give full information to women about all options [32].

By late pregnancy the women in our study appeared to be more aware of their options and the right to choose and many had chosen midwifery-led options with the apparent support of their midwives, but a number of women commented on their own lack of knowledge early in pregnancy. What is striking in our study, unlike the women in the study by Barber et al. from ten years ago [11], is that the midwife no longer appeared to be the most important source of information. Many women in our study had sought out information from other sources rather than being provided with information through a single channel by their midwife; some had to ask questions in order to get information about their options, and others had come across relevant information by chance. Friends and family, [20, 30, 33–35], word of mouth [33, 35] and women’s personal experiences of birth [19, 27, 29, 33, 36–39] were important influences on women’s decisions, as has been found previously but the Internet appears to have become a much more important source of information than has been reported in previous studies.

Internet use for pregnancy related information seeking is a global phenomenon [40, 41] and appears to be relatively common in the UK. A recent national maternity survey in England found that around three-quarters of women used online sources – predominantly NHS websites - for information relating to pregnancy and childbirth [42]. As we saw in our study, women use the internet for a variety of purposes, including for validating information, empowering themselves, sharing experiences and assisting decision-making, [43]. However, the internet is not necessarily women’s preferred source of information [44, 45]. Previous studies have found that it is important for midwives to initiate conversations about choice of birth place [19, 20, 29] and our findings do not suggest that this has changed. It is notable that many of the women in our study appear to have sought information that guidelines recommend should be provided by midwives [2].

As has been found in previous studies, our findings indicate that information given by midwives early in pregnancy was sometimes felt to be superficial and too rushed [19, 31] and women found it unhelpful to be given leaflets with inadequate time for discussion [11, 31]. A number of our study participants felt that decisions were taken too soon and that they would have valued time to reflect on their choices. Some of our participants had found out about options at antenatal classes, which are typically taken in the third trimester, a point at which decisions have generally been taken about the place of birth.

Overall our findings suggest that choice might be enhanced by avoiding taking decisions about where to give birth at a fixed time point and allowing options and decisions to be reviewed throughout pregnancy. This chimes with a recent systematic review exploring midwives’ experiences which concluded that the place of birth discussion needed to be taken at a time when “…women are ready to engage with this information, rather than automatically being given [the information] at the booking visit when they may be overloaded with information and not yet ready to consider their [place of birth] options” [46]. The review further found that midwives were not confident having place of birth discussions with women and that societal norms also affect midwives as well as women. So promoting birth in a non-OU setting as normal “needs to be facilitated to change the current culture whereby women who choose non-OU births are often perceived to be risk-takers” [46].

Following the publication of Birthplace and the revised NICE guidelines, local NHS initiatives have been developed to facilitate evidence-based conversations between midwives and women about place of birth. Some focus on providing information in a user-friendly paper format (Tracey Cooper, personal communication, 9 January 2017) or have focused on developing training and support materials for midwives (Beck Taylor, personal communication, 7 December 2016). The Portsmouth Birth Place choice project [11, 47] demonstrated that the uptake of midwifery-led birth settings could be increased through high-level organisational commitment and by implementing specific measures including training and support for midwives, to ensure that women are given evidence-based information and guidance. The Portsmouth project resulted in the development of the My Birthplace app [48] which is now available for women and midwives to use in a number of areas. Initiatives such as these have the potential to increase the proportion of women who have access to informed choice about place of birth.

### Strengths and limitations

A key strength of our study is that it was conducted after the 2014 update of the NICE intrapartum care guidelines and thus in a period when planned birth in a midwifery-led setting was recommended as the most suitable option for low risk women. It is a national study that focused on inclusivity in its sampling and innovative methodological approach using online focus groups. This enabled us to include 69 women with a good range of socio-economic groups, parity and geographical spread across England. As well as those who were very active in using the Internet to find out information, we also had others (for example Bridget, focus group 8, planning OU birth; Clara Focus group 6, opt-out AMU areas) who didn’t search for information about their choices on the internet but followed the experience of family and friends.

However, while using an online approach as a mechanism to increase the range and inclusivity of our sample, we must be mindful that our participants were therefore likely to be Internet users, and skilled at online research, and were possibly more likely to be ‘active choosers’ (women who seek the information that they need to exercise choice) rather than ‘acceptors’ (who tend to ‘go with the flow’) [49]. Our findings may not therefore fully capture the experiences and needs of this latter group. In seeking to include women from lower socio-economic groups we worked with a women’s support group in east London to recruit women for our face to face group. We were therefore unable to pre-screen the women as we did for the online groups, and were unable to collect the same biographical information about them. While the online focus groups afforded many advantages, we are also aware that there were limitations in the depth of data we were able to capture using this approach. The live chats were fast paced with women often ‘talking’ (typing) over each other, and there was a slight time lag. Therefore the ability to pick up on, and explore, particular threads of conversation during these live events was limited, in comparison to a face to face focus group or indeed a one to one interview. While questions were posed during the week on the message boards, women did not necessarily respond to them.

### Implications for policy and practice

While for our participants, midwives were not for the most part the main source of information about their birth place options, midwives continue to be an important ‘sounding board’ for women, most of whom want to be able to talk about their options with their midwife. Maternity care providers therefore need to ensure that midwives are appropriately trained, have access to user-friendly support materials and have adequate time, so that all women, not just those who will actively seek out information for themselves, are aware of their options and can make informed decisions. Consideration should be given to how best to make reliable locally-tailored information readily available to women, covering options outside the woman’s local NHS trust where appropriate. This information might be online or in a format which midwives can use directly with women. Midwives may need to be more aware of existing online resources that women may access, e.g. Which? Birth Choice [http://www.which.co.uk/birth-choice/]. The NHS Choices website, which currently does not give clear information about the types of services (e.g. OU, AMU, FMU) available at each hospital, might benefit from enhancement. Irrespective of the information source, women should have the opportunity to explore and consider their options in their own time, and revisit these later in pregnancy with their midwife. While it is important that women are given information about birth place options early in their pregnancy, our findings support an approach in which women’s decision-making is an ongoing process, not a one-off irrevocable choice.

Further research is required to determine the best way of ensuring that women are aware that they have the right to choose, are given appropriate information about their options and have the opportunity to have their questions answered by their midwife. New approaches, including the development of digital technologies, may emerge from the areas now designated as NHS ‘Maternity Choice and Personalisation Pioneers’ [50] which will be testing ways of improving choice and personalisation for women accessing maternity services.

## Conclusions

Our findings suggest that women may be encountering fewer overt obstacles to exercising choice than in the past but women do not consistently receive information about birthplace options from their midwife at a time and in a format they find helpful. Women in some areas continue to have a restricted range of options. Women’s ability to choose a birth place that meets their needs and preferences could probably be facilitated by presenting choice early in pregnancy together with information, and possibly guidance on online sources where additional reliable information can be found, but it may be best to defer *decision-making* about birth place until the woman has had time to consider and discuss her options. Approaches should take into consideration that, although women may access other sources of information, women continue to want to be able to discuss their preferences and options with their midwife.

## Additional files

Additional file 1: Focus group sample discussion guide. (DOCX 20 kb)

Additional file 2: Characteristics of focus groups participants. (DOCX 25 kb)

Additional file 3: NVIVO codes used for analysis. (DOCX 20 kb)

## Abbreviations

AIMS: Association for Improvements in the Maternity Services
AMU: Alongside Midwifery Unit
DIPEx: Database of individual patient experiences
FMU: Freestanding Midwifery Unit
MU: Midwifery Unit
NCT: National childbirth trust
NHS: National Health Service
NICE: The National Institute for Health and Care Excellence
NPEU: National Perinatal Epidemiology Unit
OSOP: One sheet of paper
OU: Obstetric unit
