# Supplementary material for: Birthplace choices: what are the information needs of women when choosing where to give birth in England? A qualitative study using online and face to face focus groups
Source: BMC Pregnancy Childbirth. 2018 Jan 8;18:12. doi: 10.1186/s12884-017-1601-4 (PMC5759241; doi:10.1186/s12884-017-1601-4)
Supplement: Supplementary file 1 — Focus group sample discussion guide. (DOCX 20 kb) [file 12884_2017_1601_MOESM1_ESM.docx]

## Birthplace Choices Online Focus Groups

**Sample discussion guide – group 1 (planning a homebirth)**

### LIVE CHAT

**Questions for live chat (moderated by LH, 60 minutes, day 1)**

**Welcome everyone, we are going to start by asking everyone to introduce themselves and tell us how many weeks pregnant they are. Whereabouts in the country do you live/first baby?**

**We want to understand how women decide where they are going to give birth. Why you would like to give birth in a particular place and how you make choices based on your local options.**

Can you tell us where you are choosing to have your baby?

Why are you choosing to have your baby there? What did you like about it?

Who did you talk to when making your decision?

How did you gather information about your options of where to give birth?

[What information did your midwife give you about birthplace options?](http://birthplacechoices1.co.uk/pt/What-information-did-your-midwife-give-you-about-birthplace-options-6.24.2015/discussion.htm)

When did you first talk about options with your midwife in your pregnancy? Did you feel you needed to make your decisions at the first meeting or are you given time to think about your choices?

Did you feel supported in your decision?

Does the area that you live in offer the choices that you would like?

Did you consider postnatal care when deciding where to give birth?

What matters to you?

Why are you choosing an AMU, would you go to a FMU if there was one nearby?

Were you offered the option of a homebirth?

**We have various questions that we would like to discuss in the discussion boards through the next 6 days, but please feel free to add your own if you think there are aspects of birthplace and choice that we have not covered.**

**Questions for discussion boards**

Q. How did you gather information about your options of where to give birth?

Q. How did you assess the quality of different options in your area?

Q. Did you visit any maternity units?

Q. Did your midwife discuss choice of where to give birth at any time during your pregnancy?

Q. How did your midwife or doctor react to your preferred place to give birth?

Q. Did you feel supported in your decision?

Q. Did you feel you had enough information?

Q. How much of an influence did talking to other people have on the decision that you made?

Q. Were you told why a midwifery unit or a homebirth might be a good choice?

Q. What choices do you think you have available to you in your area?

Q. What is your understanding of what the various options provide –Obstetric Unit (OU)/Alongside maternity unit (AMU)/Freestanding maternity unit (FMU) and homebirth?

Q. Were you offered a choice of where to give birth?

Q. Were you able to choose the setting you wanted?

Q. Were there any options that you would have liked that weren’t available to you?

Q. What factors were important to you when you were choosing your place of birth?

Q. Did you consider postnatal care at all when making your choices?

Q. Are there factors that we’ve not included that were important to you?

Q. Did anything else influence you?

Q. Why does your chosen place to give birth feel safe to you?

Q. (Not for homebirth) Did the size of the unit influence you?

Q. (For Homebirth) Did you consider how likely you could transfer to a maternity unit if you needed to during labour?

Q. Does an alongside maternity unit (AMU) feel safer than a freestanding maternity unit (FMU) ? Why? (

Q. Does an Obstetric unit (OU) feel safer than an alongside maternity unit (AMU)? Why?

Q. Have you changed your mind since making your initial decision? If so, what made you change your mind? Were you able to switch or have you encountered any problems?

Q. Is there anything you would have liked to have been done differently when making your decision?

Q. What would you need locally to feel supported in having a homebirth?

Q. In an ideal world, what would you like your local maternity service to provide?
