# Supplementary material for: Birthplace choices: what are the information needs of women when choosing where to give birth in England? A qualitative study using online and face to face focus groups
Source: BMC Pregnancy Childbirth. 2018 Jan 8;18:12. doi: 10.1186/s12884-017-1601-4 (PMC5759241; doi:10.1186/s12884-017-1601-4)
Supplement: Supplementary file 2 — Characteristics of focus groups participants. (DOCX 25 kb) [file 12884_2017_1601_MOESM2_ESM.docx]

**Characteristics of focus group participants including distance to maternity units in the woman’s area**

| **GROUP/ NAME** | **AGE** | **IMD QUINTILE *** | **PARITY** | **TYPE OF AREA/ REGION** | **OPTION INTERESTED IN/ CHOSE** | **OPTIONS AVAILABLE** |  |
| --- | --- | --- | --- | --- | --- | --- | --- |
| **Group 1 – Planning a homebirth** | | | | |  |  | |
| Ann | 32 | 3rd | Primip | City, North East | Homebirth | OU & AMU 2 miles, FMU 7 miles, OU 5 miles |  |
| Anita | 32 | 1st | Multip | Town, South West | Homebirth | FMU 7 miles, AMU 11 miles, OU 11 miles, FMU 26 miles |  |
| Angela | 34 | 1st | Multip | City, South West | Homebirth | OU & AMU 0-3 miles, AMU 4 miles, OU 4 miles |  |
| Amy | 28 | 5th | Primip | City, East Midlands | Homebirth | OU 0-3 miles, OU & AMU 4 miles, OU 15 miles, FMU 21 miles |  |
| Amelia | 33 | 5th | Primip | London | Homebirth | OU & AMU 0-3 miles, 2 x OU & AMU 4 miles |  |
| Alison | 30 | 1st | Multip | Town, South East | Homebirth | FMU 16 miles, OU & AMU 19 miles, FMU 18 miles |  |
| Amanda | 38 | 2nd | Multip | Town, South West | Homebirth | OU & AMU 0-3 miles, FMU 5 miles |  |
| Alexa | 28 | 4th | Primip | London | Homebirth | OU & AMU 0-3 miles, OU & AMU 0-3 miles |  |
| Abigail | 31 | 1st | Multip | Town, South East | Homebirth | OU & AMU 0-3 miles, OU 6 miles, OU & AMU 19 miles |  |
| Agnes | 30 | 2nd | Primip | Town, North West | Homebirth | OU & AMU 4 miles, FMU 7 miles, OU & AMU 11 miles |  |
| **GROUP 2 – living in area with lots of choice** | | | | |  |  | |
| Jenny | 30 | 2nd | Primip | City, North West | AMU | FMU 0-3 miles, OU & AMU 7miles,OU & AMU 8 miles, OU & AMU 9 miles |  |
| Julie | NK | 3rd | Primip | London | OU | OU 0-3 miles, 2 x OU & AMU 4 miles, OU & AMU 5 miles |  |
| Jude | 28 | 3rd | Multip | Town, East of England | AMU | OU & AMU 5 miles, OU & AMU 9 miles, FMU 17 miles |  |
| Janice | 34 | 4th | Primip | London | AMU or FMU, possibly home | OU 0-3 miles, OU & AMU 4 miles , OU &AMU 4 miles, OU &AMU 5 miles |  |
| Joyce | 26 | 2nd | Multip | Town, South West | Homebirth | OU & AMU 4 miles, FMU 6 miles, |  |
| Jackie | 28 | 3rd | Primip | Town, South West | Homebirth | OU & AMU 0-3 miles, FMU 6 miles |  |
| Joanna | 42 | 3rd | Primip | City, West Midlands | OU | OU & AMU 4 miles, FMU 6 miles |  |
| Jean | 24 | 3rd | Primip | Town, East of England | OU | FMU 0-3 miles, FMU 6 miles, OU & AMU 11 miles |  |
| **GROUP 3 – Living in area with limited choice** | | | | |  |  | |
| Rachel | 34 | 2nd | Primip | Town, South West | homebirth, possibly FMU | FMU 6 miles, OU & AMU 21 miles |  |
| Rebecca | 30 | 2nd | Primip | Town, West Midlands | homebirth, possibly AMU | OU 15 miles, OU & AMU 19 miles, OU 27 miles |  |
| Ruby | 32 | 2nd | Primip | Village, North West | OU | OU 11 miles, OU 31 miles, FMU ≥ 40 miles |  |
| Rosie | 34 | 2nd | Multip | Town, South East | homebirth | FMU 0-3 miles, OU 10 miles, OU & AMU 22 miles |  |
| Rita | 35 | 2nd | Primip | Village, South East | homebirth | OU 0-3 miles, FMU 16 miles, OU & AMU 18 miles |  |
| Roberta | 32 | 1st | Primip | Village, South East | FMU | FMU 6 miles, OU 19 miles, OU & AMU 22 miles |  |
| Rona | 38 | 3rd | Multip | Town, South West | Homebirth | FMU 6 miles, FMU 7 miles, FMU 16 miles, OU 13 miles, OU & AMU 30 miles |  |
| **GROUP 4 - First time mothers** | | | | |  |  | |
| Mandy | 31 | 1st | Primip | Town, South East | AMU | OU & AM 13 miles, FMU 16 miles, FMU 19 miles, OU 24 miles, FMU 26 miles |  |
| Maria | 33 | 5th | Primip | London | FMU | OU & AMU 0-3 miles, OU 0-3 miles, OU & AMU 6 miles, OU & AMU 0-3 miles, FMU 4 miles |  |
| Mabel | 36 | 2nd | Primip | Town, East of England | AMU (27 miles, not nearest) | OU & AMU 8 miles, FMU 19 miles, OU &AMU 19 miles, OU & AMU 27 miles |  |
| Mina | 33 | 4th | Primip | City, Yorkshire & Humber | OU/AMU | OU 4 miles, OU 6 miles, OU 8 miles, OU 9 miles, FMU 14 miles, OU &AMU 20 Miles |  |
| Mae | 27 | 1st | Primip | Village, South East | AMU or FMU | OU & AMU 8 miles, FMU 20 miles, OU 25 miles |  |
| Maggie | 32 | 5th | Primip | Town, North East | OU | OU 0-3 miles, OU & AMU 3 miles, OU 9 miles |  |
| **GROUP 5 – Living close to an FMU** | | | | |  |  | |
| Kirsten | 26 | 4th | Multip | Town, South West | FMU | FMU 0-3 miles, OU & AMU 11 miles, FMU 14 miles, OU & AMU 25 miles |  |
| Karolina | 35 | 5th | Multip | City, South West | FMU | FMU 0-3 miles, OU & AMU 11 miles, FMU 14 miles |  |
| Keira | 35 | 5th | Multip | City, South East | AMU or FMU | FMU 0-3 miles, OU & AMU 6 miles, FMU 18 miles, FMU 12 miles |  |
| Katrina | NK | 5th | Multip | Town, South East | AMU or FMU | FMU 0-3 miles, OU & AMU 11 miles, FMU 13 miles, FMU 25 miles |  |
| Kylie | 27 | 1st | Multip | Town, South East | AMU | FMU 0-3 miles, OU & AMU 12 miles, FMU 16 miles |  |
| Kathryn | 34 | 2nd | Primip | City, South West | AMU | OU & AMU 0-3 miles, FMU 5 miles, OU & AMU 7 miles |  |
| Kim | 36 | 1st | Primip | Village, South East | FMU | FMU 8 miles, OU 16 miles, OU & AMU 16 miles |  |
| Kerry | 30 | 2nd | Primip | Village, South East | AMU | FMU 4 miles, OU 16 miles, OU & AMU 19 miles |  |
| Kate | 35 | 1st | Primip | Village, North West | FMU | FMU 5 miles, FMU 12 miles, OU & AMU 10 miles |  |
| **GROUP 6 - Opt out AMU areas** | | | |  |  |  | |
| Clare | 32 | 1st | Primip | City, Yorkshire & Humber | OU | OU & AMU 0-3 miles, OU 11 miles, AMU &OU 13 miles |  |
| Charlotte | 34 | 4th | Primip | City, West Midlands | AMU | OU & AMU 0-3 miles, OU 10 miles, OU 9 miles |  |
| Chloe | 39 | 3rd | Multip | City, North West | AMU | OU & AMU 0-3 miles, OU 9 miles, OU & AMU 14 miles, OU & AMU 17 miles |  |
| Courtney | 32 | 4th | Primip | City, North West | AMU | OU & AMU 4 miles, OU 11 miles, OU & AMU 15 miles |  |
| Clara | 35 | 5th | Primip | City, North West | AMU | OU & AMU 0-3 miles, OU & AMU 12 miles |  |
| Chantel | 38 | 4th | Multip | Town, East Midlands | OU | OU & AMU 11 miles, OU & AMU 13 miles, FMU 16 miles |  |
| Carmen | 29 | 2nd | Primip | City, South West | AMU | OU & AMU 7 miles, FMU 12 miles, OU 16 miles |  |
| Carrie | 31 | 4th | Primip | City, South West | AMU | OU & AMU 0-3 miles, FMU 16 miles, OU 19 miles, FMU 19 miles |  |
| Cath | 32 | NK | Primip | Village, East Midlands | AMU | OU & AMU 10 miles, OU & AMU 12 miles, FMU 14 miles |  |
| Caroline | 32 | 4th | Primip | City, North West | AMU | OU & AMU 0-3 miles, OU & AMU 13 miles, OU & AMU 19 miles |  |
| Carla | NK | 1st | Multip | City, North West | OU | OU & AMU 4 miles, OU & AM 6 miles |  |
| **GROUP 7 (face to face) – Living in disadvantaged area**** | | | | |  |  | |
|  |  | **ETHNICITY** |  |  | **BOOKED FOR BIRTH IN:** | **DISTANCE/TRAVEL TIME AS DESCRIBED BY PARTICIPANT** |  |
| Yasmin | NK | Black African | Primip | East London | labour ward (not low risk) | 15 mins drive to OU |  |
| Yana | NK | Bengali-speaking | Primip | East London | labour ward (not low risk) | 10 mins walk to OU (OU w/o AMU) |  |
| Yadavi | NK | Bengali-speaking | Multip | East London | labour ward | 10 mins drive to OU (OU w/o AMU) |  |
| Yavi | NK | Indian | Multip | East London | labour ward (not low risk) | "Close" to two OUs |  |
| Yelena | NK | Eastern European | Multip | East London | FMU | 5 mins to FMU |  |
| Yusra | NK | NK | Multip | East London | AMU | 15-20 mins drive to AMU, distance to FMU "too great" |  |
| Yihana | NK | Black African | Multip | East London | AMU | Homeless |  |
| **GROUP 8 - Planning to give birth in an OU** | | | | | |  |  |
| Brenda | 23 | 5th | Primip | City, North West | labour ward | OU & AMU 0-3 miles, FMU 6 miles, OU & AMU 4 miles |  |
| Barbara | 29 | 4th | Primip | City, North West | labour ward | OU & AMU 4 miles, OU & AMU 6 miles, OU & AMU 4 miles |  |
| Beatrice | 35 | 1st | Multip | Town, South East | labour ward or homebirth | OU & AMU 5 miles, FMU 12 miles, OU 17 miles, FMU 21 miles |  |
| Bev | 28 | 2nd | Multip | Town, South East | labour ward or homebirth | OU 0-3 miles, OU & AMU 19 miles, FMU 26 miles, |  |
| Beverley | 35 | 4th | Primip | City, South West | Labour ward | OU 4 miles, FMU 30 miles, OU 40 miles |  |
| Bobbi | 29 | 2nd | Multip | City, South West | Labour ward | OU 6 miles, FMU 27 miles, OU 37 miles |  |
| Bree | 33 | 1st | Multip | Town, South East | labour ward | OU 5 miles, OU & AMU 14 miles, OU 23 miles, FMU 22 miles |  |
| Belinda | 32 | 4th | Multip | Town, South East | labour ward | OU 4 miles, OU & AMU 19 miles, FM 21 miles |  |
| Bridget | 35 | 3rd | Multip | City, South West | labour ward | OU 0-3 miles, FMU 32miles, OU ≥ 40 miles |  |
| Bonnie | 39 | 4th | Multip | London | Labour ward | OU & AMU 0-3 miles, FM 4 miles, OU & AMU 6 miles |  |
| Betty | 25 | 2nd | Primip | Town, South West | Labour ward | OU 6 miles, FMU 33 miles, FMU 35 miles |  |
| * Index of Multiple Deprivation (IMD) quintile: 1st = most disadvantaged, 5th = most advantaged. | | | | | |  | |
| ** Participants recruited through local women's support group and not pre-screened by the researchers. | | | | | |  | |
