# Supplementary material for: Birthplace choices: what are the information needs of women when choosing where to give birth in England? A qualitative study using online and face to face focus groups
Source: BMC Pregnancy Childbirth. 2018 Jan 8;18:12. doi: 10.1186/s12884-017-1601-4 (PMC5759241; doi:10.1186/s12884-017-1601-4)
Supplement: Supplementary file 3 — NVIVO codes used for analysis. (DOCX 20 kb) [file 12884_2017_1601_MOESM3_ESM.docx]

**Birthplace choices focus groups**

**NVivo Codes**

Experience of midwives when making choice

- information given by midwives
- support from midwives for decision made
- talking about their options with their midwife

Information sources

- Books
- Family/partner’s views
- Friends and other women
- hospital tours and talks
- hospital videos
- Hospital websites
- Internet
- local antenatal and exercise classes
- Local Facebook groups
- local pregnancy support groups
- midwife or doctor or other health professional
- national baby shows
- own work experiences
- previous birth experience
- research studies and evidence

Information women need from midwives

- How information should be given
- Type of information women want from midwives
- When information should be given in pregnancy

Other people's views on women's choice

Reasons for choice

- access to medical facilities if needed
- access to One to One midwives service
- accessibility (distance to travel, parking, traffic)
- antenatal experience at hospital
- availability of facilities at time of labour
- choosing between maternity hospitals
- environment
- familiarity with unit and or hospital
- gut instinct
- hospital reputation/friends have had good or bad experiences there
- makes it easier to have someone to look after other children
- own family experience of birth
- believe it is the safest option
- pain relief options
- personal views of what birth should be like
- postnatal care and environment
- preferred method of pain relief
- previous bad experience at that hospital
- previous birth experience
- size of unit
- statistics and evidence
- to avoid medical intervention if possible
- transfer to hospital
- views on FMUs
- views on midwives capabilities to manage their birth
- views on transferring from AMU to OU
- views on what an OU is like
- when to go in to hospital during labour
- where have some control over the birth process
- Why choosing AMU not OU

Things they'd like to change about the unit they are planning to give birth in

Things would like to change about AMUs

Views on arranging a homebirth

What choice means to women

- women's knowledge of the choices available to them
- women's views of the choices available in their area
- Feelings about being induced

What it's been like making their decision

Changing views during course of pregnancy

Continuity of care from midwives

Doulas
